# Supplementary material for: Novel Mutation in the Feline GAA Gene in a Cat with Glycogen Storage Disease Type II (Pompe Disease)
Source: Animals (Basel). 2023 Apr 13;13(8):1336. doi: 10.3390/ani13081336 (PMC10135178; doi:10.3390/ani13081336)
Supplement: Supplementary file 1 [file animals-13-01336-s001.zip › animals-2318921-supplementary.pdf]

Table S1. Primers used to amplify exons and splicing regions of the feline *GAA* gene.

Exon no.	Name	Primer Sequence (5′→3′)	Length (mer)	GC (%)	T_m (ºC)	Amplicon (bp)	T_a (ºC)
1	UTR	Not sequenced					
2	fGAA-E2a-F	ATTGGCGTGAGTCCCCTGAG	20	60	55.9	344	57
	fGAA-E2a-R	AGCTGCTCTGGGACTGCCGT	20	65	57.9		
	fGAA-E2a-F2	CGGCCCTTCTCTCAGCA	17	65	57	222	58
	fGAA-E2a-R2	TCGATAAAGCCCCTCAGCT	19	50	57		
	fGAA-E2b-F	GGGCTTTATCGAGCTCGCCA	20	60	55.9	364	58
	fGAA-E2b-R	GCACGTCCAGGCGTAAGGTCA	21	62	58.3		
	fGAA-E2b-F2	GCTCCATGATTTCTTGCTG	19	45	55	219	56
	fGAA-E2b-R2	ACACTGCTCCTGGGTGA	17	58	55		
	fGAA-E2c-F	AGCTGCACAACCTGACCACCA	21	57	56.3	251	57
	fGAA-E2c-R	GACTCCCCGTGAAGGCACAA	20	60	55.9		
	fGAA-E2c-F2	CCCAACAGCCGCTTCGAC	18	63	60.8	249	59
	fGAA-E2c-R2	GGCGTAAGGTCAAGATGTCC	20	55	60.5		
3	fGAA-E3-F	CTCCTGGAGGGTGAGGAAG	20	63	55.4	328	56
	fGAA-E3-R	CAGGATCGCTGTGTATGGTG	20	55	53.8		
	fGAA-E3a-F2	CTCCTGGAGGGTGAGGAA	18	63	58.4	183	58
	fGAA-E3a-R2	CTGGAGGTCCACGCTGTA	28	55	58.4		
	fGAA-E3b-F2	CTCATCCACACTTTACAGCG	20	55	58.4	175	58
	fGAA-E3b-R2	ATCGCTGTGTATGGTGGAGT	20	55	58.4		
4	fGAA-E4-F	CGTCAGTGCATCACCGTGGTG	21	62	58.3	348	58
	fGAA-E4-R	CGTTAGCAGCCTCACCCACATG	22	59	58.6		
5	fGAA-E5-F	GTACAAGGGGCAGTGAAGACG	21	57	56.3	257	57
	fGAA-E5-R	CTGGCCTGGGAGCTCAGAAG	20	65	57.9		
6	fGAA-E6-F	AGCAGGCTGTGGATTGGTGGC	21	62	58.3	343	58
	fGAA-E6-R	CGGCATGAATGGGGAGCCTG	20	65	57.9		
	fGAA-E6-F2	CAGTATGGGGCGCAGGGAT	18	61	60.8	282	60

	fGAA-E6-R2	TCTGTGAGCGCGTGGGCA	18	61	60.8		
7	fGAA-E7-F	CTGGAAGTCGTGGGTAGGCTG	21	62	58.3	345	58
	fGAA-E7-R	ACTGCGTGTCCTGTGGCCACA	21	62	58.3		
	fGAA-E7-F2	TGCCCACGCGCTCACAGA	18	61	60.8	221	60
	fGAA-E7-R2	CAGCACAGGGACGGAGTC	18	61	60.8		
8	fGAA-E8-F	GCCAGGTGGTGGAGAACATGA	21	57	56.3	301	57
	fGAA-E8-R	TCCCTTCCCTAACAGCTGGCA	21	57	56.3		
9	fGAA-E9-F	TCTGTCCTCCGTCAACGCTG	20	60	55.9	323	56
	fGAA-E9-R	CCTGTCCTCTTCCTGAACGC	20	60	55.9		
	fGAA-E9-F2	AGTGGGTGCCTGGCTTTG	18	61	58.4	229	58
	fGAA-E9-R2	CCGTTTTCTGCCCTCTCC	18	61	58.4		
10, 11	fGAA-E10,11-F	CAGGCAGGTGAGCGAGGCT	19	68	57.6	447	56
	fGAA-E10,11-R	CTGAGTCTCCCAAACCAGAG	20	55	53.8		
	fGAA-E10-F2	GGCAGGTGAGCGAGGCT	17	71	59.8	249	58
	fGAA-E10-R2	AGACAGGCCCCGGAGGT	17	71	59.8		
	fGAA-E11-F2	AAGTGTCCCGTGGGCTGT	18	61	58.4	241	58
	fGAA-E10-R2	AGCCCTGATCTGAGCCTC	18	61	58.4		
12	fGAA-E12-F	CAGAGGAGACGATACAGGGAC	21	57	56.3	301	58
	fGAA-E12-R	TCTGGGAGTCTGTGTGCGGTC	20	62	58.3		
13	fGAA-E13-F	CGGCTCATCATCCAGGGTGGT	21	62	58.3	381	58
	fGAA-E13-R	ATGGCAGCGGCATCTCCCTGA	21	62	58.3		
	fGAA-E13-F2	TTGCTCTCGGCCCTGCCA	18	67	60.8	222	58
	fGAA-E13-R2	TGACAGGCGCTCTCACCT	18	67	58.4		
14	fGAA-E14-F	TGGCTACAACGTCTGCCGGG	20	64	57.9	441	58
	fGAA-E14-R	AACCTGTACGGCTCCTGAGGC	21	62	58.3		
	fGAA-E14-F2	GCCCAGGTGGACCTACTC	18	67	60.8	286	60
	fGAA-E14-R2	TCCGCTCTCACTGCCGGT	18	67	60.8		
15	fGAA-E15-F	AACGACCTCCACAGCCTGGTAG	22	62	58.6	454	58
	fGAA-E15-R	CCTGAGCTGCACTCTCATGGG	21	62	58.3		

	fGAA-E15-F2	TGAGAGCGGAGCCGGTCT	18	67	60.8	280	59
	fGAA-E15-R2	TGTGGCAGGACGGGGCT	17	71	59.8		
16	fGAA-E16-F	AGTTTGCTGGAAAGGCCGGCAG	22	59	58.6	491	58
	fGAA-E16-R	TCCTGGGGAGCTACCCACTTG	21	62	58.3		
17	fGAA-E17-F	CCTCTGTCCCAACGACTTGT	20	55	53.8	459	56
	fGAA-E17-R	CCTCACACGCTGTCGTCTCT	20	60	55.9		
	fGAA-E17-F2	CTGTCCCAACGACTTGTCTG	20	55	60.5	279	59
	fGAA-E17-R2	ACGGACCCTCTACTGGACA	19	58	59.5		
18	fGAA-E18-F	TCCTCCAGGGTGTCTGCCTC	20	65	57.9	427	58
	fGAA-E18-R	CCTGCTCGCTTCACTTGGCTG	21	62	58.3		
19	fGAA-E19-F	TGACCGCGGTCCCCATGCA	19	68	57.6	343	58
	fGAA-E19-R	GTCCCCGGGGTCCTTGGTA	19	68	57.6		
20	fGAA-E20-F	AACAGAGCCGGAAGCCTCCAG	21	62	58.3	326	58
	fGAA-E20-R	AACTGCTTCCGACCCTGAGCC	21	62	58.3		

T_m: Melting temperature (salt adjusted), T_a: Annealing temperature used.
